# Nestin is a marker of unipotent embryonic and adult progenitors differentiating into an epithelial cell lineage of the hair follicles

**DOI:** 10.1038/s41598-022-22427-2

**Published:** 2022-10-24

**Authors:** Yuta Baba, Saki Onishi-Sakamoto, Kaori Ide, Koji Nishifuji

**Affiliations:** 1 Cooperative Division of Veterinary Sciences, Graduate School of Agriculture, Tokyo University of Agriculture and Technology, 3-5-8 Saiwai-cho, Fuchu, Tokyo 183-8509 Japan; 2 Division of Animal Life Science, Institute of Agriculture, Tokyo University of Agriculture and Technology, 3-5-8 Saiwai-cho, Fuchu, Tokyo 183-8509 Japan

**Keywords:** Differentiation, Cells, Adult stem cells, Skin stem cells

## Abstract

Nestin is an intermediate filament protein transiently expressed in neural stem/progenitor cells. We previously demonstrated that outer root sheath (ORS) keratinocytes of adult hair follicles (HFs) in mice descend from nestin-expressing cells, despite being an epithelial cell lineage. This study determined the exact stage when nestin-expressing ORS stem/precursor cells or their descendants appear during HF morphogenesis, and whether they are present in adult HFs. Using *Nes-Cre/CAG-CAT-EGFP* mice, in which enhanced green fluorescent protein (EGFP) is expressed following Cre-based recombination driven by the nestin promoter, we found that EGFP^+^ cells appeared in the epithelial layer of embryonic HFs as early as the peg stage. EGFP^+^ cells in hair pegs were positive for keratin 14 (K14) and K5, but not vimentin, SOX2, SOX10, or S100 alpha 6. Tracing of tamoxifen-induced EGFP^+^ cells in postnatal *Nes-CreERT2/CAG-CAT-EGFP* mice revealed labeling of some isthmus HF epithelial cells in the first anagen stage. EGFP^+^ cells in adult HFs were not immunolabeled for K15, an HF multipotent stem cell marker. However, when hairs were depilated in *Nes-CreERT2/CAG-CAT-EGFP* mice to induce the anagen stage after tamoxifen injection, the majority of ORS keratinocytes in depilation-induced anagen HFs were labeled for EGFP. Our findings indicate that nestin-expressing unipotent progenitor cells capable of differentiating into ORS keratinocytes are present in HF primordia and adult HFs.

## Introduction

The hair follicle (HF) is a complex structure consisting of several layers of keratinocytes arranged in concentric circles^1^. The bulge region of the outer root sheath (ORS), the outermost layer of the HF where the arrector pili muscle attaches, is a stem cell niche for HF components^2^. Previous studies demonstrated that keratin 15 (K15)-expressing HF bulge cells are multipotent epithelial stem cells capable of differentiating into keratinocytes and sebocytes in the HF epithelia and interfollicular epidermis^3,4^.

Nestin, a class VI intermediate filament protein, is a specific marker of neural stem/progenitor cells^5^. Embryonic nestin-positive cells can differentiate into neurons and glial cells. In addition, nestin expression occurs in multiple cell types in adult tissues, such as skeletal muscle satellite cells^6^, pancreatic islets^7^, testes^8^, and the heart^9^. Moreover, studies using nestin-driven green fluorescent protein (ND-GFP) transgenic mice revealed that ND-GFP cells in the upper HF are multipotent because they can differentiate into cell lineages with characteristics of neural cells, glial cells, muscle cells, melanocytes, and keratinocytes in vitro^10–14^. In addition, recent genomic analysis during follicle morphogenesis suggested that a small amount of nestin gene is expressed in placodes and epidermal cells^15^. Previously, we reported that ORS keratinocytes in adult mice were the descendants of nestin-expressing cells, despite being an epithelial cell lineage. This study aimed to define the exact stage when nestin-expression ORS stem/precursor cells appear during HF morphogenesis. We also investigated whether such stem/precursor cells are present in adult HFs.

## Materials and methods

### Mice

The *Nes-Cre/CAG-CAT-EGFP* mouse line^16^ was generated by crossing *CAG-CAT-EGFP* mice (courtesy of Junichi Miyazaki, Osaka University, Japan), in which the chloramphenicol acetyltransferase (*CAT*) gene is flanked by two loxP sites^17^, with *Nes-Cre* mice (courtesy of Ryoichiro Kageyama, Kyoto University, Japan)^18^; both mouse strains were on a C57BL/6 background. The *Nes-CreERT2/CAG-CAT-EGFP* mouse line was generated by crossing *CAG-CAT-EGFP* mice with *Nes-CreERT2* mice (courtesy of Itaru Imayoshi and Ryoichiro Kageyama, Kyoto University, Japan), which harbor the *CreERT2* gene downstream of the nestin promoter and which are on a C57BL/6 background. *CAG-CAT-EGFP* and *Nes-CreERT2* mice were provided by the Center for Animal Resources and Development at Kumamoto University. *CAG-CAT-EGFP* and *Nes-CreERT2* mice were also provided by RIKEN BioResource Research Center through the National Bio-Resource Project of MEXT (Japan).

The Animal Research Committee and Specific Biosecurity Management Subcommittee of Tokyo University of Agriculture and Technology approved all experiments using genetically-arranged mice with approval numbers #25-70 and #29-76, respectively. All animal experiments were carried out in accordance with the Animal Research: Reporting of In Vivo Experiments (ARRIVE) guidelines^19^, and all methods were carried out in accordance with relevant guidelines and regulations. Mice were kept in cages with standard woodchip bedding in a conventional mouse room under constant room temperature (25 °C) and humidity (40%) and a 12-h light/dark cycle with ad libitum feeding/drinking.

### Administration of 4-hydroxy-tamoxifen (OHT) to *Nes-CreERT2/CAG-CAT-EGFP* mice

OHT (10 mg; Cayman Chemical; Ann Arbor, MI, USA) was dissolved in ethanol to obtain a 100 mg/mL OHT suspension, which was further dissolved in corn oil (Merck, Darmstadt, Germany) to obtain a 10 mg/mL OHT solution. Finally, a volume of solution containing 1 mg of OHT was injected intraperitoneally for 5 consecutive days into *Nes-CreERT2/CAG-CAT-EGFP* mice aged 4 weeks or 7 weeks (n = 6, 20.1 ± 1.1 g body weight).

### Tissue collection

Embryonic skin was harvested from *Nes-Cre/CAG-CAT-EGFP* mice (n = 6) at various gestational ages (embryonic days 15.5, 16.5, and 18.5) when early or advanced hair germs and hair pegs were recognized^20^. Trunk skin was also sampled from this mouse line at postnatal days 0 and 33 (P0 and P33), when lanugo and anagen HFs were recognized (n = 6)^21^. In addition, the dorsal skin of 4-week-old *Nes-CreERT2/CAG-CAT-EGFP* mice (n = 3) administered OHT for 5 consecutive days was sampled on the sixth day after the initiation of OHT administration (Fig. 3a). Finally, 7-week-old *Nes-CreERT2/CAG-CAT-EGFP* mice (n = 3) were administered OHT for 5 consecutive days, their dorsal hairs were depilated to induce anagen HF on the sixth day after the initiation of OHT administration, and their dorsal skin was sampled on the seventh day after depilation (Fig. 3a). Skin samples were fixed with 10% neutral buffered formalin, embedded in paraffin, and subjected to immunofluorescence analysis.

### Immunofluorescence analysis

Paraffin-embedded formalin-fixed skin samples were sectioned (2 μm), deparaffinized, and pretreated with 10 mM citrate buffer (pH 6.8) for antigen retrieval. Sections were then incubated with blocking buffer (5% goat serum, 3% skim milk, and 0.2% Tween 20 in phosphate-buffered saline) before incubation with the following primary antibodies: monoclonal mouse anti-GFP (1:200; clone 6AT316; Abcam, Cambridge, UK), polyclonal rabbit anti-GFP (1:200; Medical & Biological Laboratories, Nagoya, Japan), polyclonal rabbit anti-laminin (1:200; Abcam), monoclonal rabbit anti-K5 (1:200; Abcam), monoclonal mouse anti-K14 (1:200; clone LL002; Abcam), monoclonal mouse anti-K15 (1:100; clone LHK15; Abcam), monoclonal rabbit anti-vimentin (VIM) (1:200; clone EPR3776; Abcam), monoclonal rabbit anti-SOX2 (1:50; clone SP76; Abcam), monoclonal rabbit anti-SOX10 (1:50; clone EPR4007-104; Abcam), monoclonal rabbit anti-S100 alpha 6 (S100A6) (1:200; clone EPR13084-69; Abcam), and monoclonal mouse anti-trichohyalin (1:200; clone AE15). Slides were then labeled with either Alexa Fluor 488- or 546-conjugated secondary antibodies (1:400; Life Technologies, Carlsbad, CA, USA) or combinations thereof for double immunofluorescence. Nuclei were counterstained with Hoechst 33258 (1:400; Life Technologies). Slides were examined under a confocal laser-scanning microscope (LSM710NLO 2 photon, Carl Zeiss, Jena, Germany; Nikon AX, Nikon, Tokyo, Japan) and image data were captured using imaging software (ZEN, Carl Zeiss; Nikon AX R, Nikon).

### Statistical analysis

A Student’s *t*-test was performed to compare frequencies of EGFP^+^ cells in epithelial cells at peg and bulbous peg stages. Furthermore, the same test was performed to compare the ratios of EGFP^+^K14^+^ cells to K14^+^ cells between the first anagen HFs and depilation-induced anagen HFs in OHT-administered *Nes-CreERT2/CAG-CAT-EGFP* mice using Graph Pad Prism8.2.1 software (GraphPad Software, San Diego, CA, USA). A *p*-value less than 0.05 was considered statistically significant.

## Results

### EGFP^+^ cells are present in the HF epithelium from the early hair peg stage in *Nes-Cre/CAG-CAT-EGFP* mouse embryos

**Figure 1:**
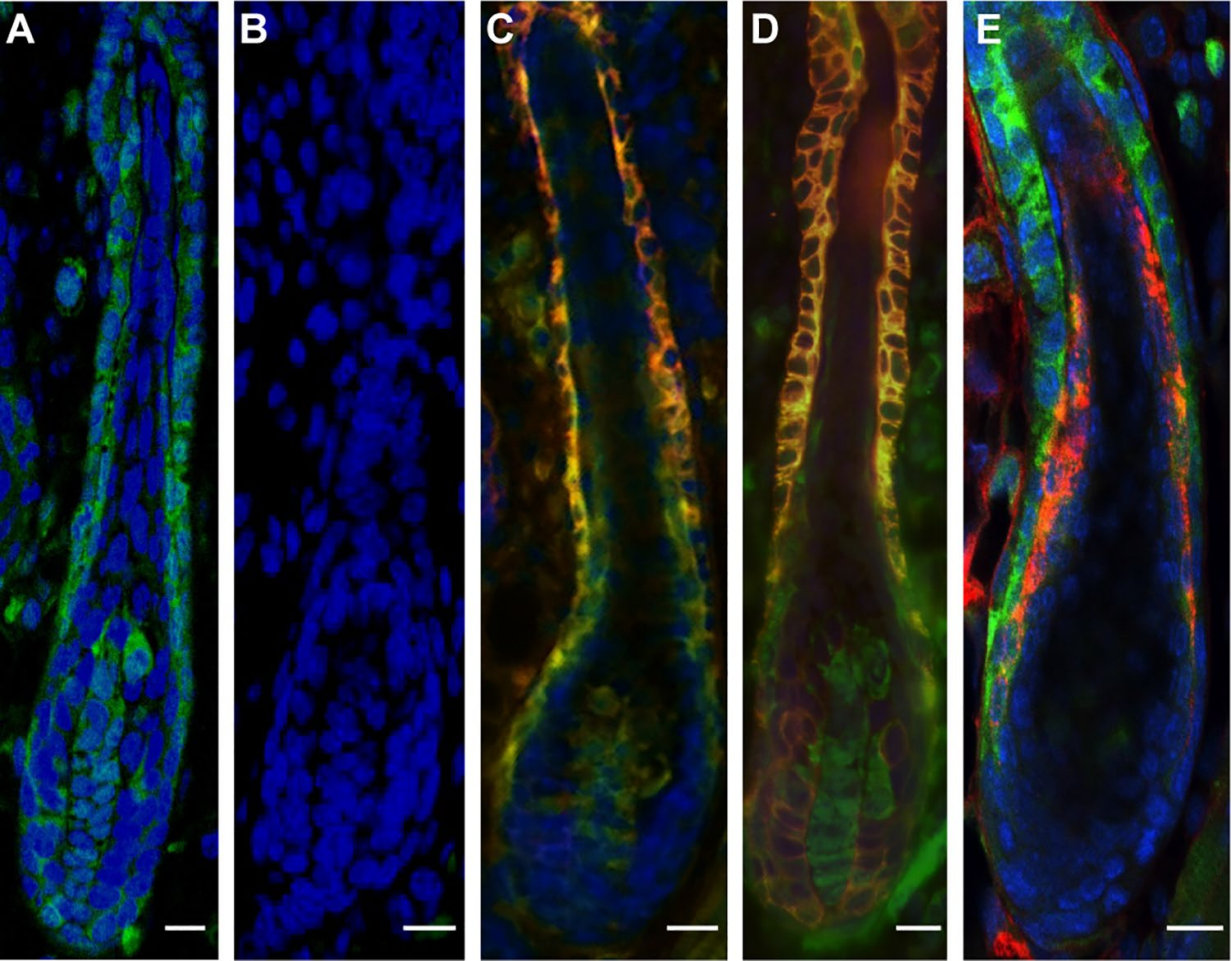
Presence of EGFP^+^ cells in the HFs of neonatal *Nes-Cre/CAG-CAT-EGFP* mice. Truncal skin of *Nes-Cre/CAG-CAT-EGFP* (**A, C, D, E**) and *CAG-CAT-EGFP* (**B**) mice was collected at P0 and immunolabeled for EGFP by monoclonal anti-GFP antibody (green). Double-immunolabeling for EGFP (green) and (**C**) K14 (red), (**D**) K5 (red), or (**E**) trichohyalin (red) in the outer layers of lanugo HFs of *Nes-Cre/CAG-CAT-EGFP* mice at P0. Nuclei were counterstained by Hoechst 33258 (blue). Scale bars, 20 μm.

We first examined the tissue distribution of EGFP^+^ cells in lanugo HFs of *Nes-Cre/CAG-CAT-EGFP* mice at the neonatal (P0) time point. Immunofluorescence analysis revealed that outer HF epithelial cells from the upper isthmus to the inferior regions were uniformly immunolabeled for EGFP (n = 3) by monoclonal and polyclonal anti-GFP antibodies (Fig. 1 and Supplementary Fig. S1). In addition, most EGFP^+^ cells were double-positive for K14 and K5, an ORS keratinocyte marker (Fig. 1), suggesting that the EGFP^+^ cells in ORS of lanugo HFs are the descendants of nestin-expressing cells. In contrast, the fluorescent intensity of EGFP in follicular keratinocytes expressing trichohyalin, an inner root sheath keratinocyte marker, was much lower than those in outer HF epithelial cell layer (Fig. 1). The ratio of EGFP^+^K14^+^ cells to K14^+^ cells in neonatal mouse HFs was 79.4% ± 9.7% (n = 6). Conversely, no EGFP^+^ cells were found in the follicular epithelium of neonatal *CAG-CAT-EGFP* mice (n = 3) (Fig. 1), indicating the specificity of EGFP immunolabeling after Cre-based recombination driven by the nestin promoter. EGFP^+^ cells were also present in spinous and granular layers, but not in the basal layer of the inter-follicular epidermis, in neonatal *Nes-Cre/CAG-CAT-EGFP* mice (data not shown).

**Figure 2:**
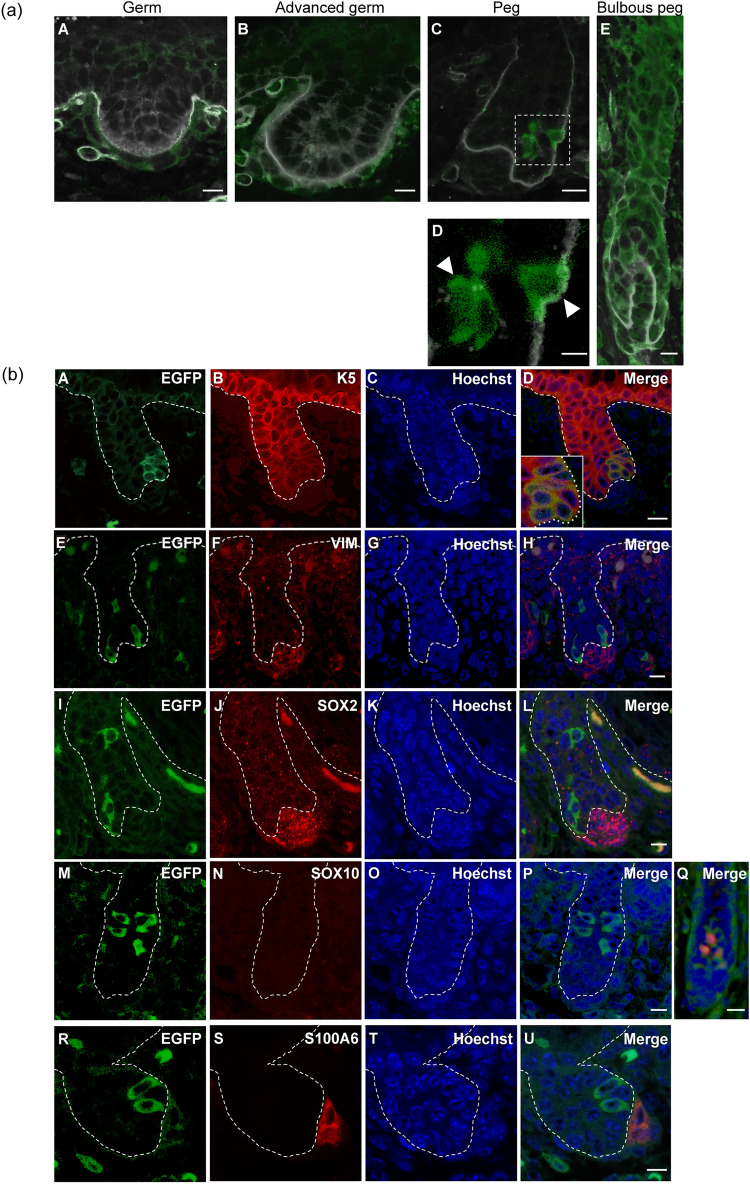
EGFP^+^ cells with characteristics of epithelial cells in *Nes-Cre/CAG-CAT-EGFP* mouse embryos during the hair peg stage*.* (**a**) Time-course analysis of EGFP expression in epithelial cells of embryonic HFs in *Nes-Cre/CAG-CAT-EGFP* mice. Skin collected at germ (**A**), advanced germ (**B**), peg (**C**, **D**), and bulbous peg (**E**) stages was subjected to immunolabeling for EGFP (green) and laminin (white). Scale bars, 10 μm (**A, B, C, E**), 5 μm (**D**). (**b**) Immunolabeling for EGFP (**A, E, I, M, R**), K5 (**B**), VIM (**F**), SOX2 (**J**), SOX10 (**N**), and S100A6 (**S**) in hair peg epithelia. Nuclei were counterstained by Hoechst 33258 (**C, G, K, O, T**). A subset of cells in anagen hair bulbs, presumably melanocytes, were immunolabeled for SOX10 (**Q**). Scale bars, 10 μm (**D, H, L, P, U**), 20 μm (**Q**).

Based on the above findings, we hypothesized that progenitor cells of ORS keratinocytes start expressing nestin during the embryonic stage. Therefore, we performed double-immunofluorescence analysis for EGFP and laminin to investigate whether EGFP^+^ cells are present in the epithelial layer during HF morphogenesis in *Nes-Cre/CAG-CAT-EGFP* mouse embryos (n = 6). HF morphogenesis in an embryo begins with placode formation and then progresses through germ, advanced germ, peg, and bulbous peg stages^1^. Although we did not observe EGFP^+^ cells in the epithelial cell layer during germ or advanced germ stages, they were present in the dermis surrounding germs in *Nes-Cre/CAG-CAT-EGFP* mice (Fig. 2a). In addition, a small subset of EGFP^+^ cells was observed in the epithelial cell layer of early hair pegs of this mouse line (Fig. 2a). During the bulbous peg stage, the majority of HF cells expressed EGFP. Furthermore, the frequency of EGFP^+^ cells in the epithelial cell layer during the bulbous peg stage (95.1% ± 4.5%, n = 6, Fig. 2a) was significantly higher than in the early hair peg stage (8.5% ± 2.3%, n = 6; Student’s *t*-test, *p* < 0.0001). Moreover, some dermal spindle cells were immunolabeled for EGFP, suggesting that these were nestin-expressing cells that eventually differentiate into papilla cells present in neonatal hair follicles or perivascular cells that make up the capillaries around hair follicles^22,23^.

### EGFP^+^ cells in *Nes-Cre/CAG-CAT-EGFP* mouse embryos exhibit characteristics of epithelial cells during the early hair peg stage

Next, we investigated whether EGFP^+^ cells in *Nes-Cre/CAG-CAT-EGFP* mouse embryos resembled epithelial or mesenchymal cell lineages during the early hair peg stage. Double-immunofluorescence analysis revealed that EGFP^+^ cells in hair peg epithelia were immunolabeled for K5. In contrast, most of these cells were not immunolabeled for VIM, although a few EGFP^+^ cells expressed VIM in a small area of cytoplasm (Fig. 2b). These findings indicate that EGFP^+^ cells in the early peg stage were of an epithelial cell lineage, although we could not exclude the possibility that they were derived from mesenchymal cells at an earlier stage of HF development. We could not determine whether EGFP^+^ cells were immunolabeled for K14 because hair peg epithelial cells were only faintly stained by the K14 antibody used in this study (data not shown). During the early hair peg stage, EGFP^+^ cells in HF epithelia in *Nes-Cre/CAG-CAT-EGFP* mouse embryos were not immunolabeled for the neural stem cell markers SOX2^24^ or S100A6^25^, or the neural crest cell marker SOX10^26^ (Fig. 2b). Dermal papilla cells in the early hair peg stage were immunolabeled for VIM, SOX2, and S100A6, indicating the specificities of the antibodies used in this study^27–29^. Moreover, EGFP^+^ cells in hair bulb epithelia of postnatal *Nes-Cre/CAG-CAT-EGFP* mice were immunolabeled for SOX10, demonstrating the specificity of the anti-SOX10 antibody to melanocytes^30^. Our study also revealed that some dermal spindle cells were immunolabeled for EGFP and SOX2, suggesting they were skin-derived precursors with multipotent differentiation potential^23,31^.

### OHT-induced EGFP^+^ cells capable of differentiating into ORS keratinocytes are present in postnatal HF isthmus epithelia of *Nes-CreERT2/CAG-CAT-EGFP* mice

**Figure 3:**
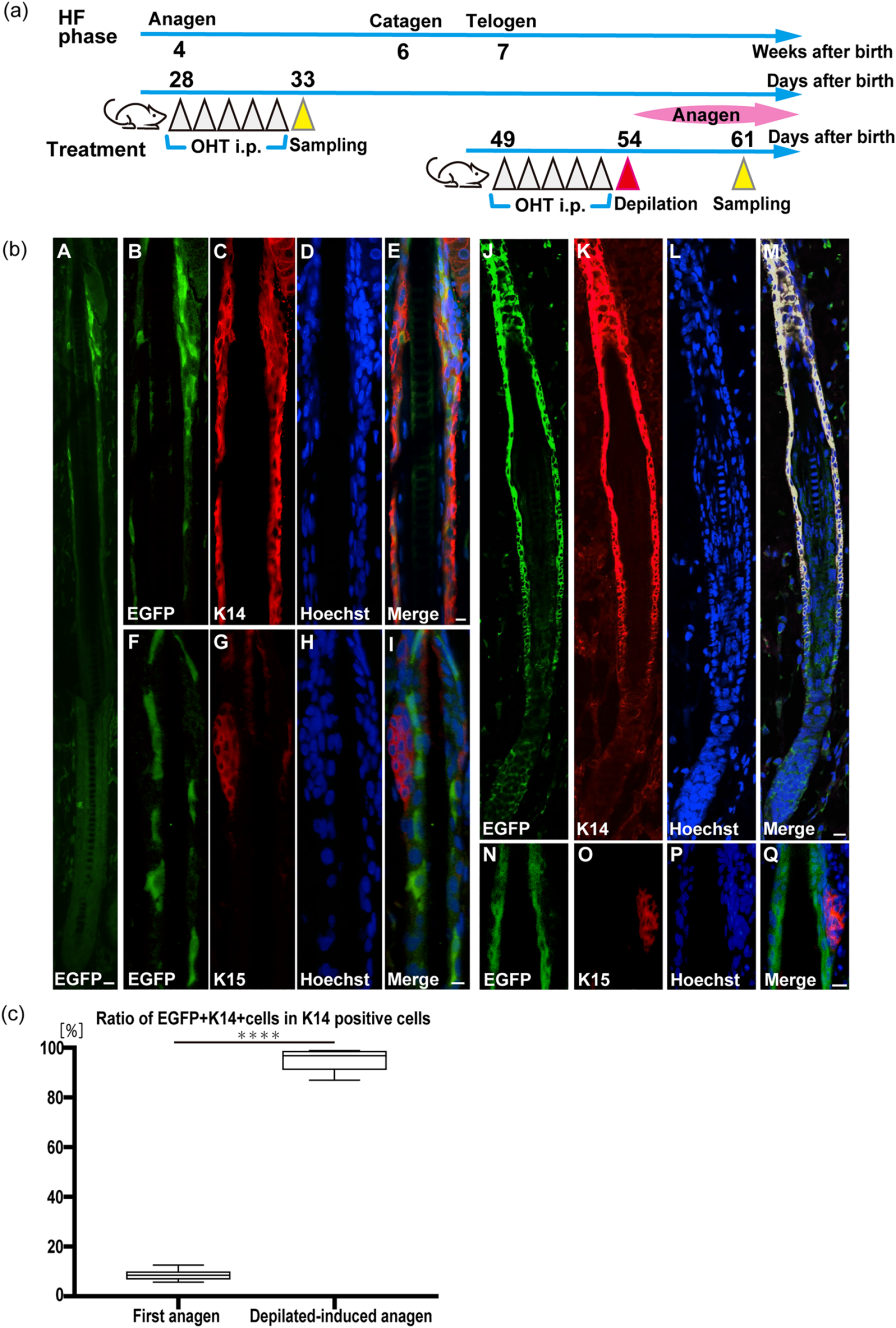
EGFP^+^ cells are present in postnatal HF epithelia and differentiate into ORS keratinocytes. (**a**) Scheme of OHT induction to express EGFP under the nestin promotor, depilation of truncal hairs, and sample collection. (**b**) Immunolabeling of EGFP (**A, B, F, J, N**), K14 (**C, K**), and K15 (**G, O**) in first-anagen HFs (**A–I**) and depilation-induced anagen HFs (**J–Q**) of *Nes-CreERT2/CAG-CAT-EGFP* mice. EGFP^+^ cells in the isthmus region of first-anagen HFs (**A, B, F**) were immunolabeled for K14 (**C**) but not K15 (**G**). The majority of EGFP^+^ cells in depilation-induced anagen HFs were immunolabeled for K14 (**K**), but EGFP^+^ cells in the isthmus region were not immunolabeled with K15 (**O**) as in the first-anagen HFs. Nuclei were counterstained by Hoechst 33258 (**D, H, L**,** P**). Scale bars, 20 μm (**A, M**), 10 μm (**E, I,** **Q**). (**c**) Comparison of frequencies of EGFP^+^K14^+^ cells in K14^+^ cells in first-anagen HFs with depilation-induced anagen HFs of OHT-administered *Nes-CreERT2/CAG-CAT-EGFP* mice (Student’s *t*-test). ****, *p* < 0.0001.

We next investigated whether nestin-expressing stem/progenitor cells capable of differentiating into ORS keratinocytes were present in adult HFs. To achieve this, we performed double-immunofluorescence analysis of EGFP and K14 expression in OHT-administered *Nes-CreERT2/CAG-CAT-EGFP* mice at 5 weeks of age, when HFs uniformly undergo their first anagen (n = 3) (Fig. 3a)^21^. We found that a small subset of K14^+^ cells in the isthmic region was immunolabeled for EGFP in OHT-administered *Nes-CreERT2/CAG-CAT-EGFP* mice (Fig. 3b). Moreover, double-immunofluorescence analysis of EGFP and K15 revealed that EGFP^+^ cells in HFs (Fig. 3b) were not immunolabeled for K15. We further examined whether EGFP^+^ cells were present in depilation-induced anagen HFs of *Nes-CreERT2/CAG-CAT-EGFP* mice (n = 3) (Fig. 3a). Our results revealed that the majority of K14^+^ cells were immunolabeled for EGFP in depilation-induced anagen HFs of OHT-administered *Nes-CreERT2/CAG-CAT-EGFP* mice. The frequency of EGFP^+^K14^+^ cells in K14^+^ cells of depilation-induced anagen HFs was 92.1% ± 4.6% (n = 6), which was significantly higher than the frequency observed in the first anagen (6.5% ± 1.8%, n = 6) (Student’s *t*-test, *p* < 0.0001; Fig. 3c). Moreover, double-immunofluorescence analysis of EGFP and K15 revealed that EGFP^+^ cells in the depilation-induced anagen HFs of OHT-administered *Nes-CreERT2/CAG-CAT-EGFP* mice were not immunolabeled for K15, as in the first anagen (Fig. 3b). These findings suggest that most of the ORS keratinocytes in depilation-induced anagen HFs are the descendants of nestin-expressing adult progenitor cells. Keratinocytes in the inter-follicular epidermis were not immunolabeled for EGFP in *Nes-Cre/CAG-CAT-EGFP* mice after 5 weeks of age (data not shown).

## Discussion

During the embryonic stage, nestin is temporarily expressed in neuroepithelial stem/progenitor cells^32^. In this study, we found that nestin-expressing progenitor cells of ORS keratinocytes appeared in the HF primordium as early as the hair peg stage. Moreover, these cells were immunolabeled for K5, suggesting that they have characteristics of epithelial progenitor cells. Co-expression of nestin and cytokeratins was previously observed in progenitor cells for lens epithelial cells^33^ and Sertoli cells^34^ in mouse embryos. However, these epithelial cells were not immunolabeled for the neural stem cell markers SOX2 or S100A6. Progenitor cells of mouse ORS keratinocytes were reported to not be derived from stem/progenitor cells expressing the neural crest cell markers Wnt1 or plasminogen activator^35^. Taken together, nestin-expressing progenitor cells of ORS keratinocytes in HF primordia are postulated to be epithelial cells that are not derived from neural crest cells. However, our results do not exclude the possibility that progenitor cells for ORS keratinocytes are derived from nestin-expressing mesenchymal cells that trans-differentiate into epithelial cells during a brief period in the early peg stage. A previous study reported weak nestin gene expression in E14.5 placodes and epidermis^15^. Conversely, our study did not observe EGFP^+^ epithelial cells in HFs before the peg stage. A possible reason for this discrepancy is that nestin expression in HF epithelia in early HF development was too weak to be detected by the immunofluorescence staining method used in this study.

This study failed to demonstrate S100A6^+^ cells in epithelia during the hair peg stage, consistent with a previous report showing an absence of S100A6^+^ cells in hair germ epithelia^29^. Conversely, S100A6^+^ cells have been identified in the bulge region of adult mouse HFs^29^. Therefore, S100A6^+^ cells may appear in HF epithelia after the peg stage. Further studies to define the exact time S100A6^+^ cells appear in HF epithelia are expected.

Our findings demonstrate that OHT-driven EGFP-expressing epithelial cells in the isthmic region of anagen HFs in 5-week-old *Nes-CreERT2/CAG-CAT-EGFP* mice are distinct from K15-expressing cells. EGFP^+^ cells are either nestin-expressing cells or their descendants in mice after 4 weeks of age. A previous study demonstrated the presence of nestin-positive, K15-negative cells in the anagen HFs from human scalp^36^. The same study also indicated that GFP^+^ cells located immediately below sebaceous glands expressed the stem cell marker CD34, but not K15, in ND-GFP mice. The stemness of EGFP^+^ cells in the isthmus of *Nes-CreERT2/CAG-CAT-EGFP* mouse HFs remains to be elucidated. Notably, an epithelial cell population co-expressing GFP and K15 was previously observed in the ORS of whisker HFs in ND-GFP mice^14^. We postulate that nestin was transiently expressed in K15^+^ cells before 4 weeks of age, or that nestin expression in K15^+^ cells of truncal HFs was too weak to be detected in the mouse line used in this study.

Our findings further imply that ORS keratinocytes are descendants of postnatal nestin-expressing progenitor cells in mouse skin. Therefore, we postulate that nestin-expressing unipotent epithelial progenitor cells for ORS keratinocytes exist in first-anagen HFs. On the basis of a comparison of the differentiation potencies between EGFP^+^ cells and K15^+^ HF bulge cells^37^, we suggest nestin-expressing cells in adult HFs are downstream of K15^+^ epithelial pluripotent stem cells. In contrast, previous studies revealed that GFP^+^ cells in ND-GFP mice exhibited multipotency in vitro^14^. Moreover, GFP^+^ cells in ND-GFP mice transdifferentiated into neural cells when subcutaneously transplanted into nude mice^38^. It is possible that some stimuli related to cell culture or transplantation triggered the pluripotency of those cells. Accordingly, the transdifferentiation capacity of nestin-expressing HF epithelial cells into distinct cell lineages in vivo remains to be elucidated.

Attempts have been made to regenerate complete HFs using totipotent or pluripotent stem cells^39^. However, to achieve more efficient HF regeneration, the detailed molecular mechanisms involved in differentiation of distinct cell lineages for each cell layer of HFs must be identified. Our study reveals a molecular marker of unipotent progenitor cells for the outermost HF cell layer in postnatal HFs. Future studies to elucidate molecular interactions necessary for the differentiation of these multipotent stem cells into downstream cells are expected.

## Supplementary Information

Supplementary Figure S1.

## Data Availability

The datasets used and/or analyzed during the current study available from the corresponding author on reasonable request.
