# Supplementary material for: Nestin is a marker of unipotent embryonic and adult progenitors differentiating into an epithelial cell lineage of the hair follicles
Source: Sci Rep. 2022 Oct 24;12:17820. doi: 10.1038/s41598-022-22427-2 (PMC9592581; doi:10.1038/s41598-022-22427-2)
Supplement: Supplementary file 1 — Supplementary Figure S1. [file 41598_2022_22427_MOESM1_ESM.docx]

Title: Nestin is a marker of unipotent embryonic and adult progenitors differentiating into an epithelial cell lineage of the hair follicles

**Supplemental Figure**

**
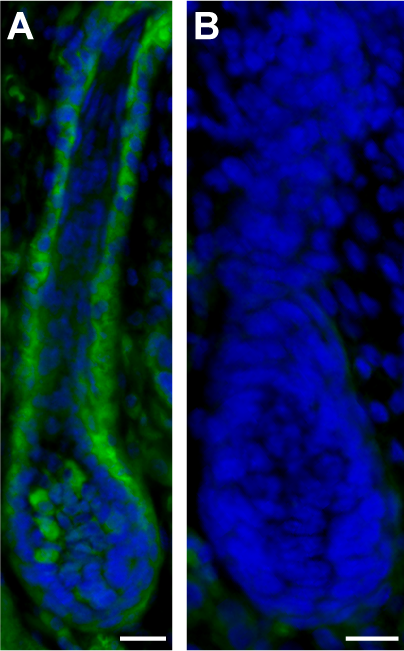
**

**Supplementary Fig. S1**. Immunolabeling of EGFP^+^ cells in the HFs of neonatal *Nes-Cre/CAG-CAT-EGFP* mice. Truncal skin of *Nes-Cre/CAG-CAT-EGFP* (A) and *CAG-CAT-EGFP* (B) mice was collected at P0 and immunolabeled for EGFP by polyclonal anti-GFP antibody (green). Nuclei were counterstained by Hoechst 33258 (blue). Scale bars, 20 μm.
